# Face and content validation of TURP and TURB simulation models: an EAU European School of Urology (ESU) Lower Urinary Tract Endoscopy Working Group Study

**DOI:** 10.1007/s00345-026-06441-x

**Published:** 2026-05-04

**Authors:** Tarik Emre Sener, Tiago Ribeiro de Oliveira, Engin Denizhan Demirkiran, Davide Perri, Sergio Pereira, Juan Pablo Caballero, Luis Osorio, Ioannis Goumas Kartalas, Rodrigo Ramos, Afonso Sousa Castro, Daniel Oliveira Reis, Marco Paciotti, Paramananthan Mariappan, Pietro Diana, Laurian Dragos, Domenico Veneziano, Chandra Shekhar Biyani, Bhaskar Somani, Evangelos Liatsikos

**Affiliations:** 1 Department of Urology, School of Medicine, Marmara University, Marmara Üniversitesi Pendik Eğitim Ve Araştırma Hastanesi, Üroloji Anabilim Dalı, 4. Kat. Fevzi Çakmak Mah, Pendik, 34890 Istanbul, Turkey; 2 Department of Urology, Armed Forces Hospital, Lisbon, Portugal; 3 Department of Urology, School of Medicine, Zonguldak Bülent Ecevit University, Zonguldak, Türkiye; 4 Department of Urology, Azienda Socio Sanitaria Territoriale Lariana, Como, Italy; 5 Department of Urology, Lisbon Medical Academic Centre, Lisbon, Portugal; 6 Department of Urology, University General Hospital, Alicante, Spain; 7 Department of Urology, Lusíadas Porto Hospital, Porto, Portugal; 8 Department of Urology, Istituto Clinico Beato Matteo, Vigevano, Italy; 9 Department of Urology, Portuguese Institute of Oncology Lisboa, Lisbon, Portugal; 10 Department of Urology, IRCCS Humanitas Research Hospital Rozzano, Milan, Italy; 11 Department of Urology, Edinburgh Bladder Cancer Surgery, Western General Hospital, University of Edinburgh, Edinburgh, UK; 12 Department of Urology, Fundacio Puigvert, Barcelona, Spain; 13 Department of Urology, Cambridge University Hospitals NHS Foundation Trust, Cambridge, UK; 14 Department of Urology, Bronxcare Health System, New York, USA; 15 Department of Urology, St James’s University Hospital, Leeds, UK; 16 Department of Urology, University Hospital Southampton NHS Foundation Trust, Southampton, UK; 17 Department of Urology, University Hospital Patras, Patras, Greece

**Keywords:** TURP, TURB, Simulation, Surgical education, Validation, Endourology training

## Abstract

**Purpose:**

To evaluate the educational validity of two bench-top simulators for Transurethral Resection of the Prostate (TURP) and Transurethral Resection of Bladder Tumor (TURB), focusing on their realism, ergonomics, and relevance for structured endourology training.

**Materials and methods:**

Fourteen expert endourologists from multiple European centers assessed both simulators during the European Association of Urology Residents Education Programme (EUREP) 2025. Face validity and content validity were evaluated using 4-point Likert questionnaires. Item-level (I-CVI) and scale-level (S-CVI/Ave) content validity indices were calculated for all items and adjusted for core procedural skills.

**Results:**

Experts rated both simulators highly for anatomical realism, tissue handling, and overall utility (mean scores > 3.5/4). The TURP simulator achieved an adjusted S-CVI/Ave of 0.92 and the TURB simulator 0.97, indicating excellent consensus on their educational adequacy for key procedural steps. Non-modeled features such as bleeding, obturator reflex, and energy modulation received low ratings, reflecting inherent limitations of bench-top simulation. Both models were considered effective for practicing instrument handling and resection depth control in a risk-free, standardized environment.

**Conclusions:**

The TURP and TURB simulators demonstrated strong face and content validity for core resection training. Their modular, non-biological, and reproducible design supports safe, structured skill acquisition and competency assessment in endourology curricula, offering a practical bridge between theoretical learning and clinical performance.

**Supplementary Information:**

The online version contains supplementary material available at 10.1007/s00345-026-06441-x.

## Introduction

Transurethral resection of the prostate (TURP) remains the gold standard surgical treatment for benign prostatic obstruction in appropriately sized prostates [1]. Despite its widespread use, TURP is a technically demanding procedure associated with a significant learning curve and potential complications such as bleeding and capsular perforation [2–4]. Similarly, transurethral resection of the bladder (TURB) remains the standard for the treatment of bladder tumors. En-bloc technique seems to provide fewer complications and higher pathological information compared to conventional resection, but with no differences in oncological outcomes [5]. Despite the fact that TURB may seem like an easy procedure, often performed by residents, 100 cases have been calculated as the absolute minimum for a resident in training to achieve acceptable oncological and surgical outcomes [6]. Notably, surgical experience (specialist vs. resident) has been shown as predictive for recurrence after TURB [7]. Traditional training methods, which rely heavily on intraoperative exposure (on-the-job training), may expose patients to unnecessary risks, suboptimal management and provide limited opportunities for deliberate practice [8].

Simulation-based training has emerged as a valuable adjunct to surgical education, offering a safe and reproducible environment for skill acquisition [9–11]. Although several endourological simulators have been developed, their ability to accurately replicate the anatomy, ergonomics, and procedural steps of TURP and TURB varies considerably [10]. Establishing the educational validity of these models is critical to ensure their effective integration into training curricula.

This study aimed to evaluate the educational validity of newly developed TURP and TURB simulators. Specifically, we assessed face validity, defined as experts’ perception of realism and training usefulness, and content validity, defined as the degree to which the simulator covers the essential steps of the procedure [12]. By combining these two complementary approaches, we sought to determine the extent to which the model can serve as a reliable tool for teaching the core skills required for TURP and TURB procedures.

## Methods

### Study design

We conducted face and content validation of two bench-top endourological simulators representing TURP and TURB. Expert endourologists independently completed structured questionnaires for each model. Experts were defined as consultant urologists with over 200 TURPs and TURBs performed prior to this study.

As there is no universally accepted numerical cutoff for defining expertise in simulator validation studies, the threshold of > 200 TURP and TURBT procedures was chosen pragmatically and conservatively to ensure that expert participants were clearly beyond the learning curve and had substantial independent procedural experience [3, 6].

A total of 14 experts were involved in the assessment of the simulators. The study was conducted during the European Association of Urology Residents Education Programme (EUREP) 2025 in Prague, Czech Republic).

### Training model

The TUR Trainer by Samed GmbH (Dresden, Germany) is a dedicated simulation device developed to provide a realistic platform for the practice of both TURP and TURB. The Samed training box is modular, and can be used both for prostate and bladder tumor resection training.

**Fig. 1:**
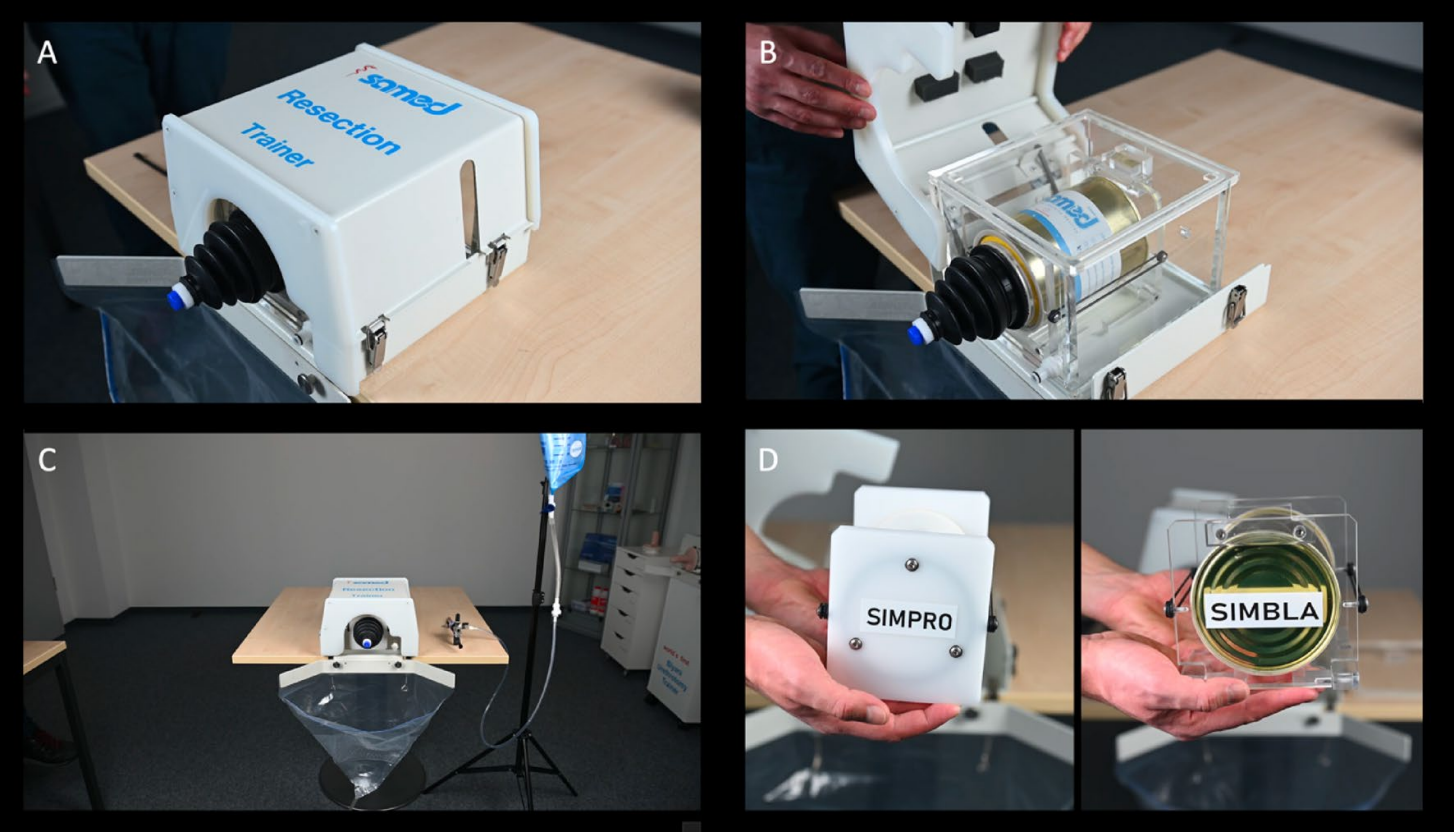
The Samed TUR Trainer (**A–C**) designed for repeated practice of transurethral resection techniques. The attached bag (**C**) is used to collect irrigation outflow generated during the simulated resection. The replaceable prostate and bladder modules (**D**) are called SIMPRO (prostate) and SIMBLA (bladder), respectively

The trainer consists of a compact anatomical box that replicates the bladder. A sealed construction with an elastic urethral entry port permits the insertion of a standard resectoscope, and a transparent collection bag facilitates fluid management and evacuation of the resected tissue. The system can be opened using secure metal clasps, which allow for the straightforward replacement of training modules (Fig. 1).

### TURP model

The replaceable prostate model is supplied in airtight sealed tin, ensuring both hygiene and convenience in storage. Each tin contains a specially engineered semisynthetic protein matrix that closely mimics the resistance and resectability of human prostatic tissue. A single tin provides a complete resection training session and can be quickly exchanged for a new one, when needed.

**Fig. 2:**
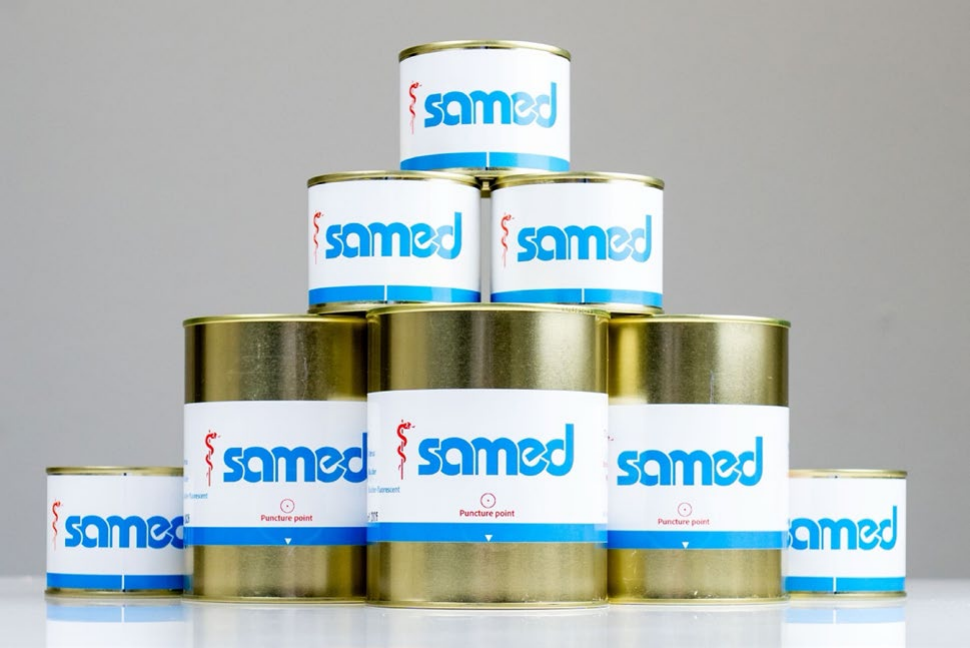
The Samed replaceable prostate and bladder modules supplied in sealed cans, designed for repeated practice of transurethral resection techniques

The prostate training model was constructed based on a 3D schematic drawing that reproduces the internal anatomy of the gland. It incorporates a central urethral lumen with a clearly defined verumontanum, as well as two lateral lobes and a small median lobe, making it suitable for realistic resection practice. This design allows the user to identify key anatomical landmarks and replicate the steps of clinical TURP in a standardized setting (Fig. 2).

The prostate models are available in different sizes, corresponding to approximate gland weights of 40 g, 60 g, and 80 g, in order to simulate varying anatomical conditions. In the present study, the 40 g model was used. The prostate models are compatible with resections using bipolar resectoscopes.

### TURB model

The replaceable bladder model is supplied in airtight sealed cans, ensuring hygiene and convenient storage. Each model is manufactured from a semisynthetic protein matrix engineered to closely mimic the resistance and resectability of human bladder and tumor tissue. A single can supports a complete resection training session for two trainees and can be rapidly exchanged for a new model when needed. This design enables efficient turnover during training sessions while maintaining consistent tissue-like properties.

**Fig. 3:**
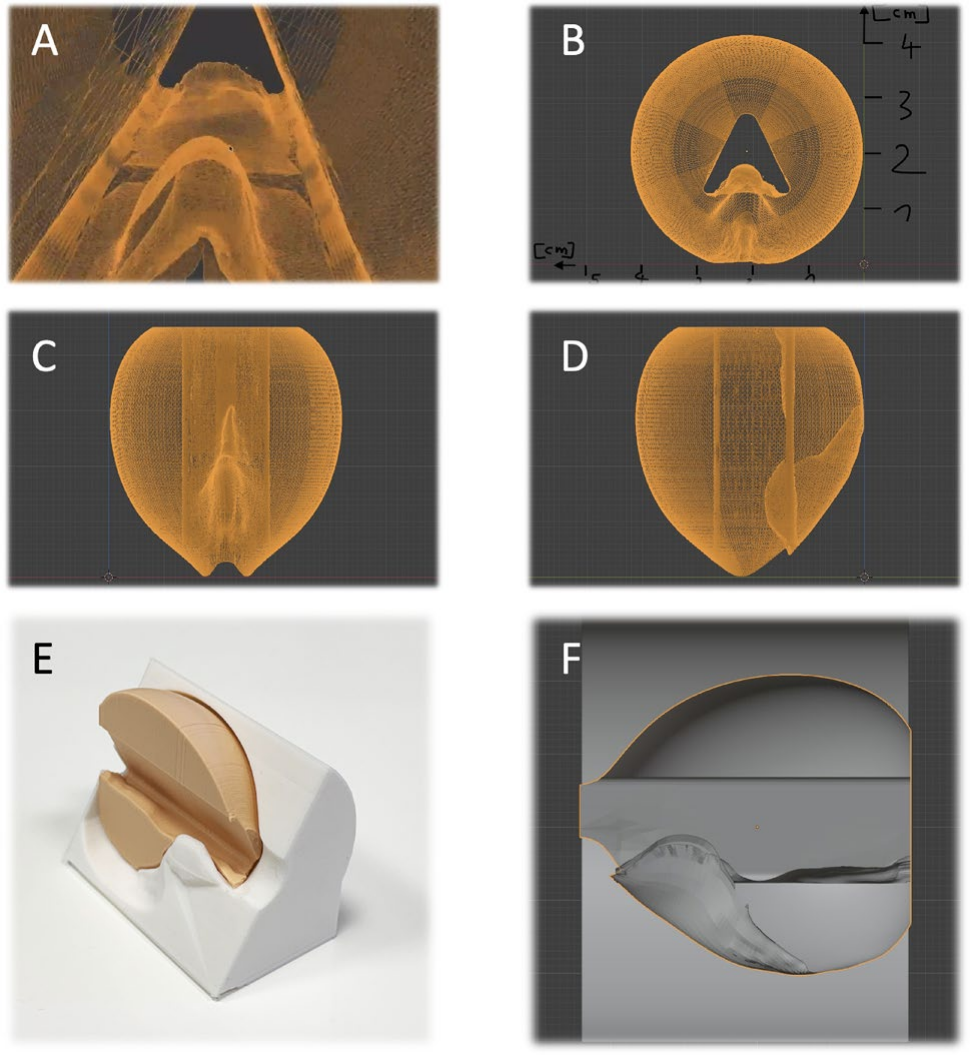
Schematic drawing of the TURP training model. The 3D renderings show the central urethral passage in endoscopic (**A**), coronal (**B**), axial (**C**) and sagittal (**D**, **F**) views illustrating the anatomical structure replicated for TURP training and the synthetic prostate (**E**)

**Fig. 4:**
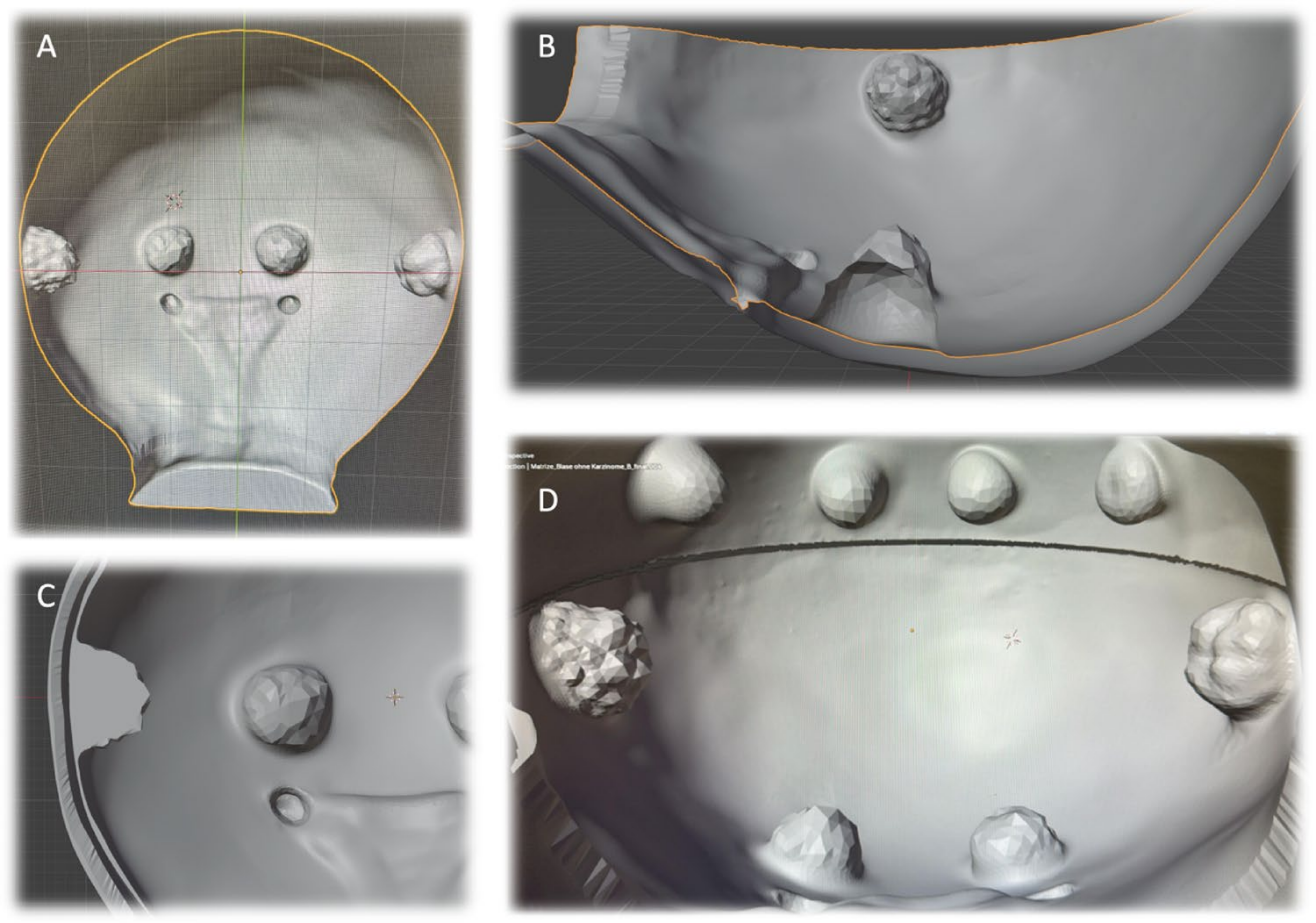
Schematic drawing of the TURB training model. The model consists of an upper and lower compartment. The lower compartment (**A**, **B**) includes the trigone with two ureteric orifices and contains four tumors (two lateral and two medial). The bladder wall is composed of 2 layers that can be distinguished from each other during resection under endoscopic view (**C**). The upper compartment also carries four tumors (D)

The TURB simulator consists of a modular bladder design with two compartments. The lower compartment incorporates the trigone and two ureteric orifices, together with four tumor nodules positioned laterally and medially on each side (Fig. 3). It is constructed with three distinct layers (mucosa, muscle, and serosa), providing higher anatomical fidelity and more realistic tactile feedback. This section is primarily dedicated to essential training and structured examination. The upper compartment contains four additional tumors and is composed of two layers, allowing repetitive resection practice while reducing production costs (Fig. 4). The structural difference between the two parts (three-layered lower vs. two-layered upper) balances realism with feasibility, reserving anatomically detailed sections for critical tasks.

### Face and content validity questionnaires

**Fig. 5:**
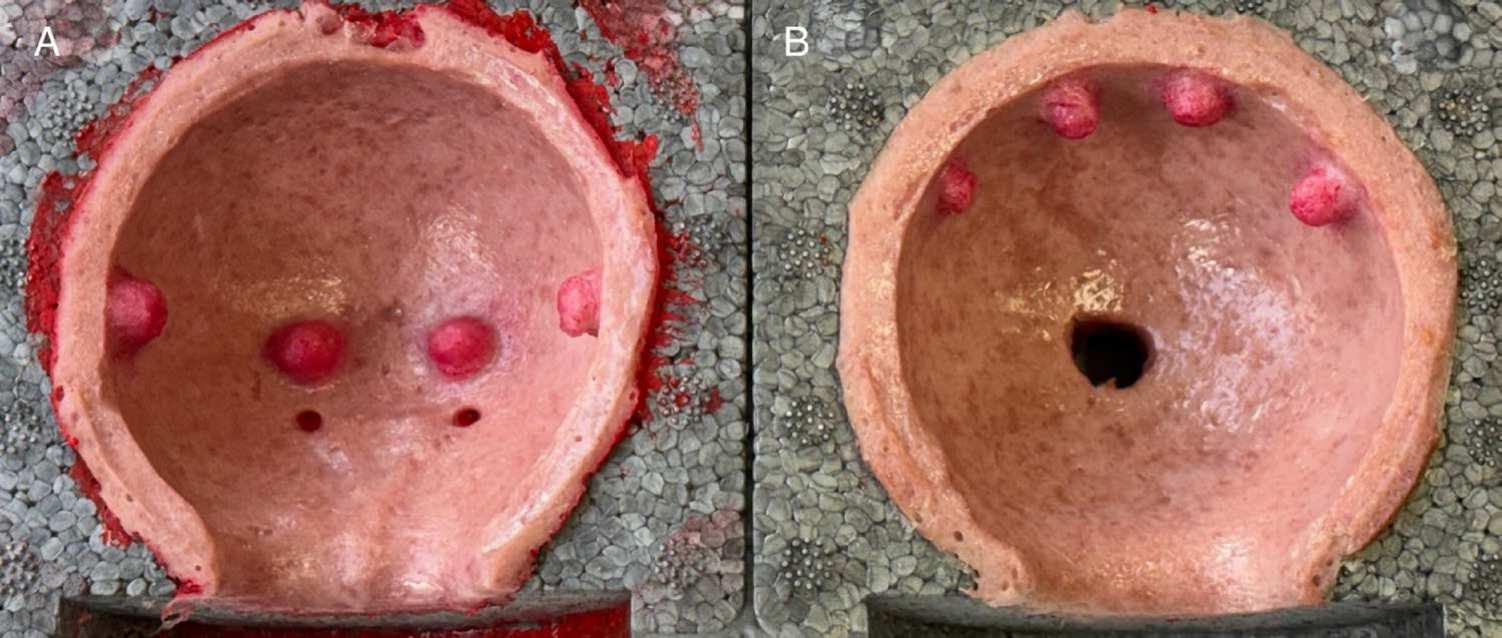
Picture of the printed TURB training model. The model consists of a lower (**A**) and upper (**B**) compartment. The lower compartment includes the trigone with two ureteric orifices and contains four tumors (two lateral and two medial). The upper compartment also carries four tumors

The Face and Content Validity Questionnaires were developed by the European School of Urology (ESU) Lower Urinary Tract Endoscopy Working Group as part of the standard validation framework for transurethral training models (see Fig. 5).

### Face validity

Face validity captured perceived realism and training usefulness on a 4-point Likert scale (1 = Strongly Disagree, 2 = Disagree, 3 = Agree, 4 = Strongly Agree). The items addressed anatomical realism, tissue handling, endoscopic environment, visibility, ergonomics, procedural steps, intraoperative challenges, and overall utility. Responses were converted into numerical values [1–4]. Descriptive statistics were calculated for all items. Higher scores indicated stronger agreement among experts regarding realism and training usefulness. The face validity results are presented descriptively as means with standard deviations, minimum, and maximum values for each item.

### Content validity

Content validity was assessed by item relevance using a separate questionnaire in which experts rated the relevance of simulator features on a 4-point scale (1 = Not Relevant, 2 = Somewhat Relevant, 3 = Quite Relevant, 4 = Highly Relevant). Content validity is reported using the Item-level Content Validity Index (I-CVI) values for each item and the overall scale-level Content Validity Index (S-CVI)/Ave, compared with established thresholds to determine adequacy [13].

Item-level content validity refers to the relevance of each individual questionnaire item. For each item, I-CVI was calculated as the proportion of experts rating the item as 3 or 4, divided by the total number of experts:

$$ {\mathrm{I}} - {\mathrm{CVI}} = {\text{Number of experts rating 3 or 4 }}/{\text{ Total number of experts}} $$

The I-CVI provides a quantitative measure of the degree of agreement among experts that a specific item is relevant to TURP training.

Scale-level content validity refers to the overall adequacy of the questionnaire or simulator assessment as a whole. The S-CVI/Ave summarizes the overall adequacy of the instrument by indicating whether, as a whole, the simulator’s features are judged to be relevant for training. To evaluate the overall scale, the Scale-level Content Validity Index (S-CVI/Ave) was calculated as the mean of all I-CVI values: S-CVI_Ave_ = ∑I-CVI / Number of items.

Thresholds recommended in the literature were applied: an I-CVI of ≥ 0.78 was considered acceptable for individual items when the number of raters was ten or more [14], while an S-CVI/Ave of ≥ 0.80 was considered indicative of adequate content validity for the scale as a whole [13].

In this study, the I-CVI was used to determine expert agreement on specific simulator features, whereas the S-CVI/Ave was used to reflect the overall educational relevance of the simulator for TURP or TURB training.

### Domain-specific analysis

While overall content validity reflects expert ratings of all items, certain features assessed in the questionnaire are not realistically represented in bench-top simulators. Including these items in the calculation of the S-CVI/Ave may underestimate the educational value of the model [15, 16].

To address this, a domain-specific analysis was performed. The items were divided into two groups: core procedural skills that the simulators were designed to teach, and advanced or non-modeled features that fell outside the simulator’s capabilities (Table 1).

**Table 1:** Domain Mapping of Items for TURP and TURB Content Validation

Domain	TURP Items (Survey-based)	TURB Items (Survey-based)
Core Skills (included in Adjusted S-CVI/Ave)	Anatomical identification (verumontanum, lobes)	Identification of tumor location and appearance Handling and orientation of cystoscope/resectoscope
	Initiation and sequence of resection	Tumor resection with loop movement and depth control
	Loop control and depth management	Simulation of different tumor sizes and positions (lateral wall, dome, trigone)
	Chip evacuation and visibility	Evacuation and retrieval of tumor chips
	Completeness of resection	Tumor base resection and muscle sampling
		Completeness of tumor resection Overall coverage of critical steps Distinguishing novice vs advanced learners Overall usefulness for TURB training
Advanced or Non-Modeled Features (excluded from Adjusted S-CVI/Ave)	Obturator reflex	Ureteric orifice identification
	Intraoperative bleeding	Intraoperative bleeding
	Energy modulation	Obturator reflex
	Ureteric orifice identification	Energy settings adjustment

For the adjusted analysis, only the core skills items were included in the denominator, and an adjusted S-CVI/Ave was calculated. This approach allowed us to report both the conventional S-CVI/Ave (All Items) and the Adjusted S-CVI/Ave (Core Skills).

Presenting both indices provides a balanced assessment, highlighting the limitations of the models while accurately reflecting their validity as training tools for essential steps of TURP and TURB.

## Results

### TURP simulator

#### Face validity

Fourteen experts completed the face validity questionnaire for the TURP simulator. The mean rating for the model’s usefulness in teaching handling and ergonomics of the resectoscope was 3.86 ± 0.36, and for practicing procedural steps such as resection of the median and lateral lobes was 3.86 ± 0.36. The mean scores for anatomical realism, tissue handling, and endoscopic environment realism were 3.50 ± 0.52, 3.64 ± 0.50, and 3.71 ± 0.47, respectively. Visibility and access received a mean score of 3.29 ± 0.73. The lowest ratings were for intraoperative bleeding and obturator reflex (mean 1.07 ± 0.27) (Table 2).

**Table 2:** Summary of face and content validity results for the TURP simulator

Domain	Result
*Face validity – overall item score range*	1.07–3.86
*Face validity – mean score across all items*	3.39
Highest-rated face validity items	Handling/ergonomics of the resectoscope (3.86); practice of key TURP steps including median and lateral lobe resection (3.86); willingness to use for resident/fellow training (3.79); simulation of the endoscopic environment (3.71)
Intermediate face validity items	Tissue handling realism (3.64); overall realism of the simulation (3.64); sequence of steps reflecting actual TURP (3.57); anatomical realism (3.50); visibility/access comparable to live TURP (3.29)
Lowest-rated face validity item	Simulation of common intraoperative challenges such as bleeding and obturator reflex (1.07)
*Content validity – S-CVI/Ave (all 16 items)*	0.71
*Content validity – Adjusted S-CVI/Ave (core TURP skills, 12 items)*	0.92
Highest-rated content validity items (I-CVI = 1.00)	Identification of the verumontanum; initiation of resection at the appropriate anatomical location; controlled loop movement for effective tissue resection; resection of both median and lateral lobes; overall coverage of critical TURP steps; overall usefulness for TURP training
Other high-rated content validity items	Handling/orientation of the resectoscope (0.93); depth control to avoid capsular perforation (0.93); evacuation/retrieval of chips (0.93); assessment of resection completeness (0.93); distinction between normal tissue and adenoma (0.86)
Lowest-rated content validity items	Identification of ureteric orifices (0.14); identification of the external sphincter (0.43); management of intraoperative bleeding (0.14); observation/management of obturator reflex (0.00); use/adjustment of monopolar or bipolar energy settings (0.00)

#### Content validity

Item-level content validity indices (I-CVI) were 1.00 for anatomical identification of the verumontanum, 1.00 for initiation of resection, 1.00 for loop control, 0.93 for depth management, 0.93 for chip evacuation, 0.93 for completeness assessment, and 1.00 for overall coverage of critical steps. I-CVI values for intraoperative bleeding, obturator reflex, and energy setting adjustment were 0.14, 0.00, and 0.00, respectively. The S-CVI/Ave for all 16 items was 0.71, and the Adjusted S-CVI/Ave for the 12 core skill items was 0.92 (Table 2 ).

**Table 3:** Summary of face and content validity results for the TURB simulator

Domain	Result
*Face validity – overall item score range*	3.07–3.71
*Face validity – mean score across all items*	3.51
Highest-rated face validity items	Bladder geometry supporting handling/ergonomics of the resectoscope (3.71); suitability for training basic TURB skills (3.71); replication of visibility/distortion under endoscopic optics (3.64); visual feedback during resection (3.64); suitability for TURB competency evaluation (3.64)
Intermediate face validity items	Sequence of steps reflecting actual TURB (3.57); anatomical appearance of the bladder (3.50); positioning of tumors allowing realistic maneuvers (3.50); tissue handling properties (3.50); size/orientation of tumor nodules (3.43)
Lowest-rated face validity items	Interior bladder surface texture (3.21); location of ureteral orifices and bladder neck (3.07)
*Content validity – S-CVI/Ave (all items)*	0.81
*Content validity – Adjusted S-CVI/Ave (core skills)*	0.97
Highest-rated content validity items (I-CVI = 1.00)	Identification of tumor location and appearance; handling/orientation of cystoscope and resectoscope; tumor resection with appropriate loop movement and depth control; simulation of tumor base resection and muscle sampling; assessment of completeness of resection; overall coverage of critical TURB steps; overall usefulness for TURB training
Other high-rated content validity items	Identification of ureteric orifices (0.86); simulation of different tumor sizes and positions (0.93); evacuation/retrieval of tissue fragments (0.93); distinction between superficial and deeper resection layers (0.86)
Lowest-rated content validity items	Management of intraoperative bleeding (0.29); simulation/observation of obturator reflex (0.14); use/adjustment of monopolar or bipolar energy settings (0.29)

### TURB simulator

#### Face validity

Fourteen experts evaluated the TURB simulator. Mean scores were 3.71 ± 0.47 for bladder geometry supporting resectoscope ergonomics, 3.71 ± 0.47 for suitability in training basic TURB skills, and 3.64 ± 0.63 for visual feedback during resection. The mean ratings for anatomical appearance of the bladder, tumor positioning, and tissue handling were 3.50 ± 0.52, 3.50 ± 0.65, and 3.50 ± 0.52, respectively. Ureteric orifice and bladder neck location received a mean score of 3.07 ± 0.73, and bladder surface texture 3.21 ± 0.80 (Table 3 ).

**Table 4:** Overview of previously described TUR training simulators

Study	Procedure	Simulator type	Validated domains	Main strengths	Main limitations
Biswas 2020 Am J Clin Exp Urol	TURP	Apple-based physical model	Face, content, construct	Innovative, low-cost, accessible for basic practice	Limited anatomical fidelity; unable to simulate important complications realistically
Brewin 2014 J Surg Educ	TURP	Bench-top physical trainer (Bristol TURP Trainer)	Face, content, construct	Useful for structured training; experts outperformed novices on objective measures	Some aspects were perceived as less realistic
Sweet 2004 J Urol	TURP	Virtual reality simulator	Face, content, construct	Included bleeding, irrigation control, haptic feedback, and objective metrics	Predictive validity was not established
Hou 2017 J Surg Educ	TURP	Tissue-based model (restructured porcine kidney in latex bladder/urethra)	Face, content, construct	High anatomical realism; electrosurgical conductivity; realistic tissue handling	No bleeding simulation; requires tissue preparation/preservation; limited reusability; regulatory restrictions may apply
De Vries 2016 J Endourol	TURB	Bench-top simulator (Simbla TURB)	Face, content, construct	Useful for early skills acquisition and eye–hand coordination; large validation cohort	Could not replicate bleeding
Moore 2022 Can Urol Assoc J	TURB	Virtual reality simulator (Simbionix)	Content validity; internal consistency	Replicated key tasks such as tumor resection, bleeding control, and loop management; high interrater reliability	Limited modularity for isolated repetitive skills practice
Teoh 2019 World J Urol	TURB / ERBT	Tissue-based porcine bladder model	Face, content, construct	Realistic anatomy and tactile feedback; suitable for piecemeal and en-bloc resection training	Biological variability; biohazard handling; limited reusability
Yao 2024 BMC Surg	ERBT learning curve	Ex vivo porcine model	Learning curve evaluation	Useful for repetitive en-bloc resection practice; enabled learning curve assessment	Thin bladder wall; absence of perivesical fat; no bleeding or obturator reflex simulation
Neumann 2019 Eur Urol Focus	TURB	Virtual reality simulator (Uro-Trainer)	Training effect evaluation	Improved procedure duration, scope handling, and safety-related performance in novices	Transfer to clinical practice remained uncertain
Schulz 2019 J Surg Educ	TURB	Virtual reality simulator (Uro-Trainer)	Face, content, construct	Differentiated expertise levels; improved resectoscope handling and trainee confidence	Tissue realism and cost-effectiveness were questioned
Berridge 2021 World J Urol	TURP & TURB	Tissue-based vs virtual reality comparison	Comparative user evaluation	Physical model rated more realistic; VR model better simulated bleeding and hemostasis	Physical model lacked bleeding simulation; VR model had suboptimal haptic feedback

#### Content validity

The I-CVI values were 1.00 for tumor identification and appearance, 1.00 for resection technique and depth control, 1.00 for tumor base sampling, 1.00 for completeness assessment, 1.00 for overall training usefulness, 1.00 for handling and orientation of the cystoscope/resectoscope, 0.93 for simulation of tumor positions, and 0.93 for chip evacuation. The ability to distinguish between novice and advanced learners had an I-CVI of 0.86. Values for intraoperative bleeding, obturator reflex, and energy adjustment were 0.29, 0.14, and 0.29, respectively. The S-CVI/Ave for all items was 0.81, and the Adjusted S-CVI/Ave for core skills was 0.97 (Table 4).

### Comparative summary

Across both simulators, all experts completed the face and content validity assessments. Mean scores for modeled procedural features were consistently above 3 on the 4-point face validity scale. Non-modeled items, including intraoperative bleeding and obturator reflex, had the lowest ratings. Adjusted S-CVI/Ave values exceeded 0.90 for both simulators, indicating high inter-expert agreement for core procedural items.

## Discussion

This study evaluated the face and content validity of TURP and TURB simulators using expert assessments. Overall, both models were perceived as realistic, ergonomically sound, and well aligned with essential procedural steps and learning objectives.

Face validity results indicated that experts rated the simulator highly in terms of anatomical realism, tissue handling, endoscopic environment, and overall training utility. The model was particularly valuable for practicing key procedural steps such as median and lateral lobe resection, resectoscope ergonomics, and controlled loop movement. These findings suggest that the simulator is well suited for early-stage training, allowing residents to gain confidence in the basic technical components of TURP in a risk-free environment. The only area with consistently poor ratings was the simulation of intraoperative challenges, such as bleeding and obturator reflex, which the model does not reproduce. This highlights an inherent limitation that should be acknowledged when defining its role within a broader training curriculum.

The content validity analysis provided further insights. While the overall S-CVI/Ave across all items was 0.71, reflecting low agreement on features absent from the model, a domain-specific analysis focusing solely on core TURP skills yielded an adjusted S-CVI/Ave of 0.92. This demonstrates excellent expert consensus on the relevance of the simulator for fundamental procedural training. Features such as initiation of resection, loop control, depth awareness, chip evacuation, and completeness assessment were considered highly relevant, confirming the model’s suitability for structured skills training. Conversely, items related to energy modulation, management of bleeding, and obturator reflex were rated poorly, reinforcing that these aspects should not be considered training objectives of the current simulator.

These results align with previous reports on endourological training models, where simulators often achieve strong validation for basic skills but fall short in reproducing advanced intraoperative events. The distinction between validated and non-validated domains is crucial, as it ensures that training objectives are appropriately matched to the simulator’s capabilities [17]. Within a competency-based curriculum, this model can serve as an effective tool for initial skill acquisition, complementing other teaching modalities such as video-based instruction, cadaveric training, and supervised operating room exposure.

Various simulators have been described for TURP training, reflecting different balances between realism, accessibility, and standardization (Table 4). Biswas et al. presented an innovative low-cost apple-based model with demonstrated face, content, and construct validity, although its anatomical fidelity and ability to reproduce complications were limited [18]. Brewin et al. validated the Bristol TURP Trainer and showed that it was a useful educational tool with experts outperforming novices, despite some concerns regarding realism in selected aspects [19]. Sweet et al. reported a virtual reality TURP simulator with face, content, and construct validity, incorporating bleeding, irrigation control, and haptic feedback, thereby offering a more advanced simulation environment, although predictive validity remained unconfirmed [20]. Hou et al. developed a tissue-based porcine kidney model mounted in a latex bladder and urethra, which was rated highly for anatomical realism and electrosurgical conductivity, but still lacked bleeding simulation and required tissue preparation and preservation [21]. Taken together, these studies show that TURP simulators can provide meaningful training across different platforms, while each model carries practical trade-offs. In this context, our TURP simulator was designed to provide a clean, modular, reproducible, and standardized platform for repeated practice of essential resection steps within a structured curriculum.

Similarly, a range of simulators has been described for TURB training, including bench-top, virtual reality, and tissue-based models (Table 4). De Vries et al. validated the Simbla TURB simulator in a large cohort and demonstrated its usefulness particularly for early skills acquisition and eye–hand coordination, although the inability to replicate bleeding remained a notable limitation [22]. Moore et al. showed that the Simbionix virtual reality simulator had good content validity and internal consistency for key procedural steps such as tumor resection, bleeding control, and loop management, while modular repetitive skills training remained limited [23]. Teoh et al. and Yao et al. studied porcine models for piecemeal and en-bloc bladder tumor resection, highlighting realistic anatomy and tactile feedback, but also limitations related to biological variability, tissue handling, and the absence of important intraoperative events such as bleeding or obturator reflex [24, 25]. Neumann et al. and Schulz et al. further supported the role of virtual reality platforms such as the Uro-Trainer for improving handling, safety-related performance, and discrimination between different experience levels, although concerns remained regarding tissue realism, cost-effectiveness, and transfer to clinical practice [26, 27]. In addition, Berridge et al. showed that tissue-based and virtual reality simulators each have distinct advantages, with physical models offering greater realism and virtual systems better reproducing bleeding and hemostasis [28]. Overall, these findings support simulation as a valuable component of TURB education while emphasizing the trade-offs between realism, reusability, cost, and standardization. Our TURB simulator was therefore designed as a reusable and modular bench-top model focused on essential procedural skills and structured repeated practice.

Our study has several strengths, including the use of structured, validated methodologies for both face and content validation, as well as the participation of an expert panel with extensive endourological experience. By incorporating two distinct models, we were able to evaluate the training tools for both TURP and TURB, thereby addressing two of the most common procedures in endoscopic urology. The standardized design and domain-specific analyses strengthen the reliability of the findings and provide a clear framework for educational use. Limitations include the relatively small sample size and the absence of construct validity testing, which would be necessary to confirm each simulator’s ability to discriminate between novice and expert performance. In addition, neither model currently replicates complication management, such as bleeding or obturator reflex, which remains a major unmet need in resection simulation. Future research should explore modifications to address these aspects and investigate predictive validity through transfer-of-training studies.

The authors acknowledge the valuable collaboration of the expert endourologists who participated in the validation process and provided feedback for the refinement of the simulators. Although trainee feedback was not in the scope of this study, initial impressions have been positive and will be examined in future studies addressing educational impact and construct validity of these simulators.

## Conclusion

The evaluated TURP and TURB simulators demonstrated strong face and content validity for teaching core procedural skills. Their modular, non-biological design allows standardized, hygienic, and repeatable training in key steps such as anatomical orientation, resection technique, instrument handling depth, awareness, chip evacuation, and completeness assessment. While they do not replicate intraoperative complications or energy modulation, expert consensus confirms their value in early skill acquisition and structured assessment in endourology training programs.

## Supplementary Information

Below is the link to the electronic supplementary material.

Supplementary Material 1

Supplementary Material 2

Supplementary Material 3

Supplementary Material 4

Supplementary video. The video shows the complete set-up of the TransUrethral Resection simulator and the endoscopic view of the complete TURP and TURB procedures

## Data Availability

The data that support the findings of this study are not openly available due to reasons of sensitivity and are available from the corresponding author upon reasonable request.
