# Supplementary material for: Face and content validation of TURP and TURB simulation models: an EAU European School of Urology (ESU) Lower Urinary Tract Endoscopy Working Group Study
Source: World J Urol. 2026 May 4;44(1):339. doi: 10.1007/s00345-026-06441-x (PMC13139298; doi:10.1007/s00345-026-06441-x)
Supplement: Supplementary file 1 — Supplementary Material 1 [file 345_2026_6441_MOESM1_ESM.docx]

Supplementary Table 1. Face Validity Results for TURP (n = 14). The table below summarizes the descriptive statistics for each face validity item, including the number of expert responses, mean score, standard deviation, minimum, and maximum values. Ratings were given on a 4-point Likert scale (1 = Strongly Disagree, 4 = Strongly Agree).

| Item | Mean | SD | Min | Max |
| --- | --- | --- | --- | --- |
| The anatomical realism of the model is satisfactory. | 3.50 | 0.52 | 3 | 4 |
| The tissue handling properties feel realistic compared to actual TURP procedures. | 3.64 | 0.50 | 3 | 4 |
| The model realistically simulates the endoscopic environment of TURP. | 3.71 | 0.47 | 3 | 4 |
| Visibility and access within the model are comparable to live TURP surgery. | 3.29 | 0.73 | 2 | 4 |
| The model is useful for teaching handling and ergonomics of the resectoscope. | 3.86 | 0.36 | 3 | 4 |
| The model allows practice of key TURP steps, such as resection of median and lateral lobes. | 3.86 | 0.36 | 3 | 4 |
| The sequence of steps in the model reflects actual TURP procedures. | 3.57 | 0.51 | 3 | 4 |
| The model simulates common intraoperative challenges, such as bleeding and obturator reflex. | 1.07 | 0.27 | 1 | 2 |
| I would consider using this model for training residents or fellows. | 3.79 | 0.43 | 3 | 4 |
| Overall, the model provides a realistic simulation of TURP. | 3.64 | 0.50 | 3 | 4 |
