# Supplementary material for: Face and content validation of TURP and TURB simulation models: an EAU European School of Urology (ESU) Lower Urinary Tract Endoscopy Working Group Study
Source: World J Urol. 2026 May 4;44(1):339. doi: 10.1007/s00345-026-06441-x (PMC13139298; doi:10.1007/s00345-026-06441-x)
Supplement: Supplementary file 2 — Supplementary Material 2 [file 345_2026_6441_MOESM2_ESM.docx]

Supplementary Table 3. Face Validity Results for TURB (n = 14) The table below summarizes the descriptive statistics for each face validity item, including the number of expert responses, mean score, standard deviation, minimum, and maximum values. Ratings were given on a 4-point Likert scale (1 = Strongly Disagree, 4 = Strongly Agree).

| Item | Mean | SD | Min | Max |
| --- | --- | --- | --- | --- |
| The overall anatomical appearance of the bladder is realistic. | 3.50 | 0.52 | 3 | 4 |
| The size and orientation of the tumor nodules are appropriate. | 3.43 | 0.65 | 2 | 4 |
| The location of the ureteral orifices and bladder neck is realistic. | 3.07 | 0.73 | 2 | 4 |
| The interior bladder surface texture is adequately simulated. | 3.21 | 0.80 | 1 | 4 |
| The model accurately replicates visibility and distortion under endoscopic optics. | 3.64 | 0.63 | 2 | 4 |
| The positioning of tumors allows realistic resection maneuvers. | 3.50 | 0.65 | 2 | 4 |
| The bladder geometry supports teaching handling and ergonomics of the resectoscope. | 3.71 | 0.47 | 3 | 4 |
| The tissue handling properties feel realistic compared to actual TURB procedures. | 3.50 | 0.52 | 3 | 4 |
| The visual feedback during resection simulation is acceptable. | 3.64 | 0.63 | 2 | 4 |
| The sequence of steps in the model reflects actual TURB procedures. | 3.57 | 0.51 | 3 | 4 |
| This model is suitable for training basic TURB skills. | 3.71 | 0.47 | 3 | 4 |
| This model is suitable for evaluating TURB competency. | 3.64 | 0.50 | 3 | 4 |
