# Supplementary material for: Face and content validation of TURP and TURB simulation models: an EAU European School of Urology (ESU) Lower Urinary Tract Endoscopy Working Group Study
Source: World J Urol. 2026 May 4;44(1):339. doi: 10.1007/s00345-026-06441-x (PMC13139298; doi:10.1007/s00345-026-06441-x)
Supplement: Supplementary file 4 — Supplementary Material 4 [file 345_2026_6441_MOESM4_ESM.docx]

Supplementary Table 4. Content Validity Results for TURB (n = 14) The table below presents the Item-level Content Validity Index (I-CVI) for each item along with the overall Scale-level Content Validity Index (S-CVI/Ave). I-CVI values represent the proportion of experts rating the item as 'Quite Relevant' or 'Highly Relevant'. An I-CVI ≥ 0.78 was considered acceptable. The S-CVI/Ave represents the mean of all I-CVI values.

| Item | I-CVI |
| --- | --- |
| The model allows for identification of the ureteric orifices. | 0.86 |
| The model allows for identification of the tumor location and appearance. | 1.00 |
| The model supports proper handling and orientation of the cystoscope and resectoscope. | 1.00 |
| The model enables practice of tumor resection using appropriate loop movement and depth control. | 1.00 |
| The model allows for simulation of different tumor sizes and positions (lateral wall, dome, trigone). | 0.93 |
| The model supports training in the management of intraoperative bleeding. | 0.29 |
| The model allows for simulation or observation of the obturator reflex. | 0.14 |
| The model supports the use and adjustment of monopolar or bipolar energy settings. | 0.29 |
| The model enables evacuation and retrieval of resected tumor chips. | 0.93 |
| The model allows for simulation of tumor base resection, muscle sampling at the detrusor level. | 1.00 |
| The model supports assessment of completeness of tumor resection. | 1.00 |
| Overall, the model covers the critical steps necessary for learning TURB. | 1.00 |
| The model is appropriate for distinguishing between novice and advanced learners. | 0.86 |
| The model is useful for TURB training overall. | 1.00 |
| S-CVI/Ave (All Items) | 0.81 |
| Adjusted S-CVI/Ave (Core Skills) | 0.97 |
